# The *DANCR*/*miR-145-5p*/CD133 Axis Drives Osteosarcoma Stemness and Progression: Implications for Tumor Biology and Therapeutic Innovation

**DOI:** 10.3390/cells15131215

**Published:** 2026-07-03

**Authors:** Wei-Ting Cheng, Cai-Hong Yang, Jun Qi, Ya-Ping Ye, Xing Bao, Qi Mei, Jia-Chao Guo, Kai Xu

**Affiliations:** 1 Department of Oncology, Integrated TCM & Western Medicine Hospital, Tongji Medical College, Huazhong University of Science and Technology, Wuhan 430022, China; 2 Department of Orthopedics, Tongji Hospital, Tongji Medical College, Huazhong University of Science and Technology, 1095 Jiefang Avenue, Wuhan 430030, China; 3 Department of Oncology, Tongji Hospital, Tongji Medical College, Huazhong University of Science and Technology, 1095 Jiefang Avenue, Wuhan 430030, China

**Keywords:** lncRNA *DANCR*, *miR-145-5p*, osteosarcoma, cancer stem cells, CD133, autophagy, Akt/mTOR pathway

## Abstract

**Highlights:**

**What are the main findings?**

- LncRNA *DANCR* is significantly overexpressed in osteosarcoma tissues and correlates positively with stemness markers CD133, SOX2, and CD90.
- *DANCR* functions as a competing endogenous RNA (ceRNA) by sponging *miR-145-5p*, thereby mitigating the miRNA-mediated suppression of CD133.

**What are the implications of the main findings?**

- The *DANCR*/*miR-145-5p*/CD133 axis is associated with downstream PI3K/Akt/mTOR pathway activation and changes in autophagy-associated markers, thereby contributing to the expansion of osteosarcoma stem-like phenotypes.
- This intricate regulatory network provides a potentially crucial molecular target for overcoming tumor progression, metastasis, and therapy resistance in clinical osteosarcoma management.

**Abstract:**

Characterized by its highly aggressive behavior and propensity for metastasis, osteosarcoma remains a formidable clinical challenge with restricted treatment modalities. Cancer stem-like cells (CSCs) are widely recognized as central orchestrators of oncogenic progression and therapeutic intractability; however, the precise epigenetic regulations governing these processes are yet to be fully elucidated. Here, we investigated the role of the long non-coding RNA *DANCR* in regulating osteosarcoma stemness. *DANCR* expression was significantly upregulated in human osteosarcoma tissues and positively correlated with the stemness markers CD133, SOX2, and CD90. Functional assays demonstrated that *DANCR* overexpression enhanced stem-like properties, including an enriched CD133+/CD44+ cellular fraction and enhanced spheroid-forming capacity, concurrently accelerating in vitro cellular proliferation, migration, and invasive potential. In a xenograft mouse model, *DANCR* upregulation promoted in vivo tumor growth and lung metastasis. Mechanistically, dual-luciferase reporter assays and RNA immunoprecipitation (RIP) revealed that *DANCR* acts as a competing endogenous RNA (ceRNA) by sponging *miR-145-5p*, thereby facilitating the de-repression of CD133 and contributing to Akt/mTOR signaling activation. In addition, *DANCR*/*miR-145-5p* modulation was associated with changes in autophagy-associated markers. Collectively, these findings identify the *DANCR*/*miR-145-5p*/CD133 axis as a regulator of osteosarcoma stemness and progression, providing new insights into tumor biology and highlighting a potential molecular target for therapeutic investigation.

## 1. Introduction

As the predominant primary bone malignancy among adolescents, osteosarcoma accounts for approximately 60% of all diagnosed pediatric and young adult sarcomas [1]. Despite advancements in therapeutic regimens, the prognosis remains dismal; survival rates dramatically plunge from over 71.8% at 5 years for localized diseases to a mere 30.4% for cases burdened with metastatic spread [2]. Consequently, unraveling the intricate molecular mechanisms orchestrating invasion and metastasis is of paramount clinical urgency. Rooted in the cancer stem cell (CSC) theory, a distinct subpopulation termed tumor-initiating cells (TICs) is believed to harbor innate chemoresistance, fueling unyielding tumor expansion and metastatic dissemination. The remarkable heterogeneity inherent to osteosarcoma is largely attributed to CSCs, which intricately drive relapse and therapeutic failure [3]. Such resilient stem cell phenotypes are conventionally characterized by the distinct expression profile of surface antigens like CD133 and CD90, alongside core pluripotency transcription factors such as Oct3/4 and Sox2 [4,5].

Long noncoding RNAs (lncRNAs), delineated as transcripts exceeding 200 nucleotides devoid of protein-coding capacity, have emerged as pivotal master regulators. They dictate a myriad of oncogenic processes spanning tumor onset to metastatic evolution via complex epigenetic and post-transcriptional networks [6]. Functionally versatile, lncRNAs orchestrate cellular differentiation trajectories, modulate the asymmetric division crucial for CSC maintenance, and govern epithelial–mesenchymal transition (EMT) programs [7,8]. Notably, the lncRNA differentiation antagonizing non-protein-encoding RNA (*DANCR*) was initially identified as an inhibitor of epidermal differentiation [9]. Subsequent oncological investigations revealed that *DANCR* significantly amplifies stemness traits in hepatocellular carcinoma and correlates with dismal survival outcomes in colorectal malignancies [10]. Consequently, exploring the functional footprint of *DANCR* in osteosarcoma holds tremendous potential for uncovering novel prognostic markers and targeted interventions. Our initial explorations hinted that aberrant *DANCR* accumulation strongly correlates with enhanced tumor stemness and aggressive metastatic behaviors.

At the post-transcriptional level, miRNAs exert gene-silencing effects, whereas the competing endogenous RNA (ceRNA) paradigm describes a mechanism by which lncRNAs sequester target miRNAs and thereby relieve repression of downstream mRNAs. *miR-145-5p* has been reported as a tumor-suppressive miRNA in several cancer types, including osteosarcoma, where its dysregulation may be influenced by competing endogenous transcripts such as hsa_circ_0001017 [11]. Accumulated evidence indicates that *miR-145-5p* can restrain oncogenic progression by suppressing proliferation and invasion through direct regulation of E2F3 in bone tumors [12]. Moreover, it has been shown to impair stem cell characteristics and self-renewal by targeting TCTP in malignant gliomas [13]. Despite these findings, the ceRNA interplay between *miR-145-5p* and lncRNA *DANCR* within the context of osteosarcoma stemness remains incompletely defined.

Autophagy operates as an indispensable highly conserved intracellular recycling mechanism, degrading dysfunctional organelles and macromolecules to sustain nutrient equilibrium and cellular viability. Frequently hijacked by tumors, autophagy functions as a cytoprotective conduit when triggered by hostile microenvironmental stressors like metabolic starvation and hypoxia [14,15]. Molecularly, this cascade is robustly defined by the lipidation of LC3B-I to LC3B-II alongside the active lysosomal clearance of p62/SQSTM1 [16]. Mechanistically, the canonical PI3K/Akt/mTOR signaling axis serves as a predominant inhibitor of autophagic flux. Considering the putative capacity of *DANCR* to orchestrate Akt pathway components, deciphering its ultimate autophagic consequences represents a critical objective. Accordingly, the present investigation aimed to unravel the functional implications of *DANCR* in driving osteosarcoma stemness and to systematically decode the intertwined modulatory dynamics of the *DANCR*/*miR-145-5p*/CD133 axis on Akt signaling and subsequent autophagy.

## 2. Materials and Methods

### 2.1. Patient Samples and Cell Lines

A cohort consisting of 34 fresh primary osteosarcoma biopsies and their anatomically adjacent matched non-cancerous tissues was collected during radical surgical excisions at our institution between July 2019 and December 2022. To ensure molecular integrity, samples were immediately snap-frozen in liquid nitrogen within 30 min of surgical resection. The pathological diagnoses and tumor contents were strictly confirmed by two independent pathologists prior to RNA extraction. Ethical approval was granted by the Research Ethics Committee of Tongji Hospital of Tongji Medical College, Huazhong University of Science and Technology, and all procedures were conducted in strict accordance with the Declaration of Helsinki. Written informed consent was obtained from patients or their family members. Follow-up was performed every 2–3 months in the first year after surgery until March 2024. Tumor-free survival (DFS) was calculated from the date of tumor resection until tumor recurrence or metastasis, death, or last follow-up visit. Overall survival (OS) was defined as the interval between surgery and death or the last follow-up visit.

The malignant osteosarcoma cell lines HOS (less metastatic) and 143B (highly metastatic), exhibiting disparate metastatic behaviors, alongside the normal human fetal osteoblastic cell line FOB (used as a non-malignant baseline control) and the human embryonic kidney cell line HEK293T (used for dual-luciferase reporter assays due to its superior transfection efficiency), were all procured from the American Type Culture Collection (ATCC, Manassas, VA, USA). All cell lines were routinely authenticated by short tandem repeat (STR) profiling and tested for mycoplasma contamination. For cultivation, strictly complying with standard ATCC protocols, HOS and 143B cells were maintained in Eagle’s Minimum Essential Medium (EMEM, Gibco, Grand Island, NY, USA), whereas FOB and HEK293T cells were cultured in DMEM/F12 and DMEM high-glucose media (Gibco, Thermo Fisher Scientific, Grand Island, NY, USA), respectively. All media were fortified with 10% fetal bovine serum (FBS, HyClone, Cytiva, Logan, UT, USA) and 1% penicillin/streptomycin solution (100 U/mL, Gibco, Thermo Fisher Scientific, Grand Island, NY, USA). Cells were incubated at 37 °C in a stable, humidified atmosphere containing 5% CO_2_.

### 2.2. RNA Extraction and Quantitative Real-Time PCR (qRT-PCR)

Total RNA was isolated from clinical tissues or cultured cells utilizing the TRIzol reagent (Invitrogen, Carlsbad, CA, USA) following the manufacturer’s precise instructions. RNA concentration and purity were assessed using a NanoDrop 2000 spectrophotometer (Thermo Fisher Scientific, Waltham, MA, USA). Reverse transcription to synthesize cDNA was accomplished using the PrimeScript^TM^ RT Reagent Kit (TaKaRa, Tokyo, Japan) for lncRNAs and mRNAs, and a specific stem-loop primer method for miRNAs. Subsequently, qRT-PCR was executed on an ABI 7500 Fast Real-Time PCR System using SYBR Premix Ex Taq^TM^ II (TaKaRa). The relative transcript expression levels were strictly normalized against GAPDH (for *DANCR* and mRNAs) or U6 (for miRNAs) and calculated utilizing the standard 2^−ΔΔCt^ algorithmic method. All reactions were performed in triplicate.

### 2.3. Lentivirus Production, Transfection, and Stable Cell Line Construction

The lentiviral vector encoding the complete *DANCR* sequence (LV-*DANCR*) and the corresponding empty vector (LV-NC) were purchased from GeneChem Co., Ltd. (Shanghai, China). The short hairpin RNA targeting *DANCR* (sh-*DANCR*) and a non-targeting control (sh-NC) were synthesized by Shanghai SBO Medical Biotechnology Co., Ltd. (Shanghai, China) For transient transfections, the *miR-145-5p* mimic, inhibitor, and their respective negative controls (NC) were synthesized by RiboBio (Guangzhou, China). The sequence of the *miR-145-5p* inhibitor was 5′-AGGGAUUCCUGGGAAAACUGGAC-3′. Transfections were performed using Lipofectamine 3000 (Invitrogen, Thermo Fisher Scientific, Carlsbad, CA, USA) according to the manufacturer’s protocol. Briefly, cells were seeded at 2 × 10^5^ cells/well in 6-well plates. The mimic/inhibitor RNAs were introduced into the cells at a final concentration of 50 nM. Cells were harvested 48 h after transfection. For stable cell line generation, HOS and 143B cells were infected with lentiviruses and subsequently selected in the continuous presence of 2 µg/mL puromycin (Sigma-Aldrich, St. Louis, MO, USA) for two weeks to establish pure stable expression profiles.

### 2.4. Colony Formation and Wound Healing Assays

For colony formation, transfected 143B and HOS (800 cells/well) were seeded in six-well plates. The cells were cultured for about 10–14 days, then subsequently fixed utilizing 4% paraformaldehyde (Sigma-Aldrich, St. Louis, MO, USA) and visualized via 0.1% crystal violet (Sigma-Aldrich, St. Louis, MO, USA) staining for 20 min. After washing, the plates were air-dried and the total number of clones (>50 cells/clone) was counted. For wound healing assays, cells were grown to confluence in 6-well plates. A sterile 200 µL pipette tip was used to create a scratch. Images were captured at 0 and 24 h, and the wound closure area was measured using ImageJ software (version 1.53t; National Institutes of Health, Bethesda, MD, USA). The wound closure rate was calculated as [(wound area at 0 h − remaining wound area at 24 h)/wound area at 0 h] × 100%. For each biological replicate, quantification was performed using the entire matched scratch area at 0 h and 24 h, rather than by visual estimation from a single cropped representative image. Paired 0 h and 24 h images from the same wound field were analyzed under identical magnification settings, and the final values were derived from replicate-level ImageJ measurements.

### 2.5. Transwell Invasion Assay

Invasion assays were performed in a Transwell chamber (Corning Inc., Corning Inc., Corning, NY, USA) with an 8 µm pore size, pre-coated with Matrigel (BD Biosciences, Franklin Lakes, NJ, USA). A total of 200 µL transfected HOS (2 × 10^5^ cells/mL) or 143B (1 × 10^5^ cells/mL) cell suspension in serum-free medium was added to the upper chamber. The lower chamber was filled with DMEM containing 10% FBS. After 24 h, non-invading cells were removed, and invading cells on the lower surface were fixed, stained with crystal violet, and counted in five random fields under a microscope. Only cells that had invaded through the Matrigel-coated membrane and adhered to the lower membrane surface were included in the final quantification; residual cells remaining on the upper membrane surface were carefully removed with a cotton swab before fixation and staining and were not counted. After staining, the membranes were imaged from the lower surface, and five random lower-surface microscopic fields were quantified for each replicate.

### 2.6. Spheroid Formation Assay

Transfected 143B and HOS cells were inoculated into ultra-low adhesion surface 6-well plates (Corning Inc., Corning, NY, USA). The cells were re-suspended in 2 mL serum-free DMEM/F12 supplemented with 20 ng/mL EGF (PeproTech, Rocky Hill, NJ, USA), 20 ng/mL bFGF (PeproTech, Rocky Hill, NJ, USA), B27 supplement (Gibco, Thermo Fisher Scientific, Grand Island, NY, USA), and 100 U/mL penicillin/streptomycin (Gibco, NY, USA) at a density of 1 × 10^3^ cells per well. The medium was changed every other dayThe medium was changed every other day. After 10–12 days, the number and diameter of spheres (>50 µm) were measured and counted under a microscope (Leica, Wetzlar, Germany).

### 2.7. Cell Proliferation (MTS) Assay

At 24, 48, and 72 h post-transfection, HOS and 143B cells were detached using trypsin-EDTA. Cell density was adjusted, and cells were inoculated into 96-well plates (3000 cells/well) in 100 µL medium. At the indicated time points, 20 µL of CellTiter 96^®^ AQueous One Solution Reagent (Promega, Madison, WI, USA) was added to each well. After a 2-h incubation at 37 °C, the absorbance was measured directly at a wavelength of 490 nm (A490) without utilizing a stop solution, as optimization tests indicated that a 2 h incubation provided the absolute optimal linear readout range across the tested cellular densities on a Multi-scan MK3 microplate reader (Thermo Fisher Scientific).

### 2.8. Western Blotting Assays

Total protein from transfected 143B and HOS cells was isolated using RIPA lysis buffer (Beyotime Biotechnology, Shanghai, China) containing protease and phosphatase inhibitors. Protein concentration was measured using a BCA protein assay kit (Thermo Fisher Scientific, Waltham, MA, USA). Equal amounts of protein (30 µg) were separated by SDS-PAGE and transferred to a PVDF membrane (MilliporeSigma, Burlington, MA, USA). The membranes were blocked with 5% non-fat milk for 1 h and incubated overnight at 4 °C with designated primary antibodies. Specific phosphorylation sites interrogated included AKT (Ser473), mTOR (Ser2448), S6K (Thr389), and 4EBP1 (Thr37/46). Detailed information regarding the target proteins, catalog numbers, manufacturers, and specifically optimized dilution concentrations of all primary antibodies utilized is meticulously summarized in Table 1. GAPDH was used as a consistent internal loading control. After incubation with HRP-conjugated secondary antibody (1:5000, CST, Boston, MA, USA) for 1 h at room temperature, protein bands were detected using a chemiluminescent imaging system (ChemiDoc Touch, Bio-Rad, Hercules, CA, USA). Band densities were quantified using ImageJ software (version 1.53t; National Institutes of Health, Bethesda, MD, USA).

### 2.9. RNA Immunoprecipitation (RIP) and Subcellular Fractionation

To rigorously validate the ceRNA physical interaction mechanism, the RIP assay was conducted utilizing the Magna RIP^TM^ RNA-Binding Protein Immunoprecipitation Kit (Millipore, Billerica, MA, USA). Briefly, cell lysates were incubated overnight at 4 °C with magnetic beads conjugated with either an antibody directed against human Argonaute-2 (Ago2) or a non-specific control IgG. The immunoprecipitated RNA was subsequently purified and subjected to qRT-PCR. Furthermore, to ascertain the intracellular distribution of transcripts, nuclear and cytoplasmic RNA fractions were meticulously separated using the PARIS^TM^ Kit (Invitrogen) strictly adhering to the manufacturer’s protocol, prior to downstream qRT-PCR analysis.

### 2.10. Flow Cytometry and Fluorescence Activated Cell Sorting (FACS)

CD133-APC and CD44-PE antibodies were obtained from eBioscience, Thermo Fisher Scientific (San Diego, CA, USA). Approximately 1 × 10^6^ 143B and HOS cells were incubated at 4 °C for 30 min with CD133 and CD44 antibodies (1:100) in PBS containing 1% BSA. Isotype-matched IgG-APC and IgG-PE antibodies were used as negative controls. After washing with PBS, cells were analyzed by flow cytometry (CytoFLEX, Beckman Coulter, Brea, CA, USA). CytoExpert software (version 2.4; Beckman Coulter, Brea, CA, USA) was used to analyze the results. For FACS, cells were labeled with CD133-APC and CD44-PE and isolated by FACS (BD Influx, BD Biosciences, San Jose, CA, USA). Unstained cells were used as gating controls.

### 2.11. Dual-Luciferase Reporter Assays

HEK293T cells and osteosarcoma cell line 143B were seeded in 96-well plates. To validate the *DANCR*/*miR-145-5p* interaction, pmirGLO vectors containing wild-type *DANCR* (*DANCR*-WT) or a mutated binding site (*DANCR*-MUT) were constructed. The putative binding site sequence was 5′-CUGGACU-3′. Similarly, for the *miR-145-5p*/CD133 interaction, vectors with the wild-type CD133 3′-UTR (CD133-WT) or a mutated version (CD133-MUT) were created. Cells were co-transfected with 100 ng of reporter constructs and 50 nM of *miR-145-5p* mimic or mimic NC using Lipofectamine 3000. After 48 h, luciferase activity was measured using the Dual-Luciferase Reporter Assay System (Promega, USA) according to the manufacturer’s protocol. Renilla luciferase activity was normalized to firefly luciferase activity.

### 2.12. In Vivo Xenograft Model and Limiting Dilution Assays

All animal procedures were approved by the Institutional Animal Care and Use Committee of Tongji Hospital (Approval No. TJH-A20230201). Six-week-old male BALB/c nude mice were used. For the standard subcutaneous xenograft and tail-vein metastasis assays, six mice were included in each experimental group. For the in vivo limiting dilution assay, six mice were used for each injected cell dose in each group. For the subcutaneous xenograft model, 5 × 10^6^ 143B cells stably expressing LV-*DANCR* or LV-NC were suspended in 100 µL of PBS and injected subcutaneously into the right flank of the mice. Tumor volume was measured every 4 days using a caliper and calculated with the formula: Volume = (length × width^2^)/2. After 28 days, mice were euthanized, and tumors were excised, weighed, and processed for further analysis. For the metastasis model, 2 × 10^6^ cells were injected into the tail vein. After 6 weeks, mice were euthanized, and lungs were harvested, fixed in formalin, and embedded in paraffin. The number of metastatic nodules on the lung surface was counted after hematoxylin and eosin (H&E) staining. Furthermore, to conclusively assess the true intrinsic tumor-initiating capabilities, a parallel cohort for an in vivo limiting dilution assay was concurrently established and completed alongside the primary xenograft models (from 15 September 2023 to 12 November 2023). Highly purified serial dilutions (specifically 1 × 10^5^, 1 × 10^4^, and 1 × 10^3^ cells) of the stably transfected 143B cells were subcutaneously inoculated into nude mice (*n* = 6 per dose). Tumor incidence was assessed using predefined criteria and monitored over an 8-week period. The exact cancer stem cell frequencies were statistically estimated utilizing Extreme Limiting Dilution Analysis (ELDA) software [17].

### 2.13. Statistical Analysis

Data were analyzed using SPSS 21.0 statistical analysis software (SPSS, Inc., Chicago, IL, USA) and presented as mean ± standard deviation (SD) from at least three independent experiments. For comparison between two groups, a two-tailed Student’s *t*-test was performed. For multiple group comparisons, one-way analysis of variance (ANOVA) was used. Correlations were tested using Pearson’s correlation coefficient. *p* < 0.05 was considered a significant difference.

## 3. Results

### 3.1. DANCR Expression Was Positively Correlated with the Expression of Stemness Biomarkers and Enhanced Stemness of Osteosarcoma Cells

**Figure 1:**
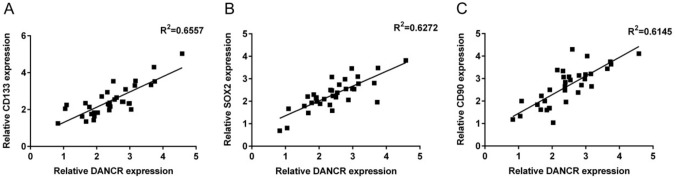
*DANCR* expression correlates with stemness markers in osteosarcoma tissues. qRT-PCR analysis showing a significant positive correlation between the mRNA levels of *DANCR* and the stem cell biomarkers CD133 ((**A**), R^2^ = 0.6557, corresponding to Pearson r = 0.81), SOX2 ((**B**), R^2^ = 0.6272, corresponding to Pearson r = 0.79), and CD90 ((**C**), R^2^ = 0.6145, corresponding to Pearson r = 0.78) in osteosarcoma tissues (*n* = 34). The solid line represents the fitted linear regression.

**Figure 2:**
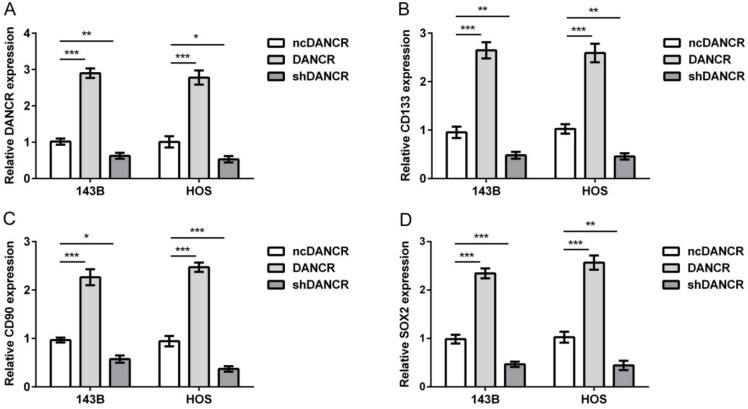
*DANCR* regulates the expression of stemness genes in osteosarcoma cells. qRT-PCR analysis of the relative mRNA expression of *DANCR* (**A**), CD133 (**B**), CD90 (**C**), and SOX2 (**D**) in 143B and HOS cells after transfection with a negative control vector (nc*DANCR*), a *DANCR* overexpression vector (*DANCR*), or a *DANCR* knockdown vector (sh*DANCR*). Data are presented as mean ± SD of three independent experiments. * *p* < 0.05, ** *p* < 0.01, *** *p* < 0.001.

The expression of *DANCR* was significantly positively correlated with the expression of stemness biomarkers CD133 (Pearson r = 0.81, *p* < 0.001) and CD90 (Pearson r = 0.78, *p* < 0.001) in osteosarcoma tissues by qRT-PCR (Figure 1A,C). *DANCR* was also positively correlated with the expression of SOX2 (Pearson r = 0.79, *p* < 0.01), as shown in Figure 1B. Consistently, expression of CD133, CD90 and SOX2 mRNA was significantly up-regulated in 143B and HOS cells overexpressing *DANCR*, compared with the control (nc*DANCR*) cells. In contrast, following transfection with sh*DANCR*, the endogenous *DANCR* expression was successfully decreased by approximately 85% compared to control cells. Consistently, the expression of these core stemness genes in the sh*DANCR* cells was significantly and proportionally inhibited, as shown in Figure 2A–D.

**Figure 3:**
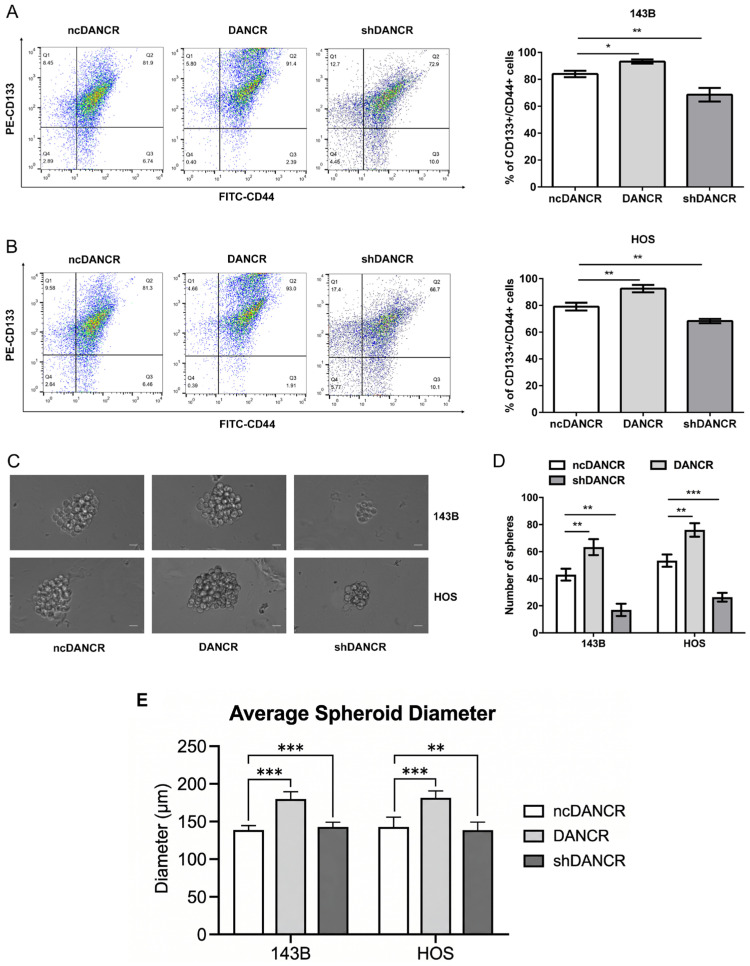
DANCR enhances stem cell-like properties in osteosarcoma. (**A**,**B**) Representative flow cytometry plots and quantification showing the percentage of CD133+/CD44+ cells in 143B (**A**) and HOS (**B**) cell lines after transfection with ncDANCR, DANCR, or shDANCR. The colors denote the experimental groups indicated in the corresponding panels. (**C**) Representative microscopic images of spheres formed by 143B and HOS cells under the indicated conditions. Scale bar = 100 μm. (**D**) Quantification of the number of spheres, showing that DANCR overexpression increases sphere formation, while knockdown decreases it. (**E**) Quantification of the average diameter of tumor spheroids, demonstrating increased size following DANCR overexpression. Data are presented as mean ± SD. * *p* < 0.05, ** *p* < 0.01, *** *p* < 0.001.

Furthermore, flow cytometry showed that *DANCR* overexpression increased the CD133+/CD44+ fraction in 143B cells from approximately 83.8% to 93.0%, whereas *DANCR* knockdown reduced this population to 68.6% (Figure 3A). A similar pattern was observed in HOS cells (Figure 3B). Because the exact absolute percentage depends on the gating strategy and cell-line background, these flow-cytometry results were interpreted together with the spheroid formation assays and in vivo limiting dilution data to support *DANCR*-associated expansion of stem-like phenotypes.

Spheroid formation assays showed that *DANCR* overexpression significantly increased both the number and average diameter of tumor spheroids, whereas *DANCR* knockdown reduced these parameters in both 143B and HOS cells (Figure 3C–E). These findings indicate that *DANCR* enhances not only stemness-related gene expression but also the functional sphere-forming capacity of osteosarcoma cells.

### 3.2. DANCR Promotes Osteosarcoma Cell Proliferation, Migration, and Invasion In Vitro

**Figure 4:**
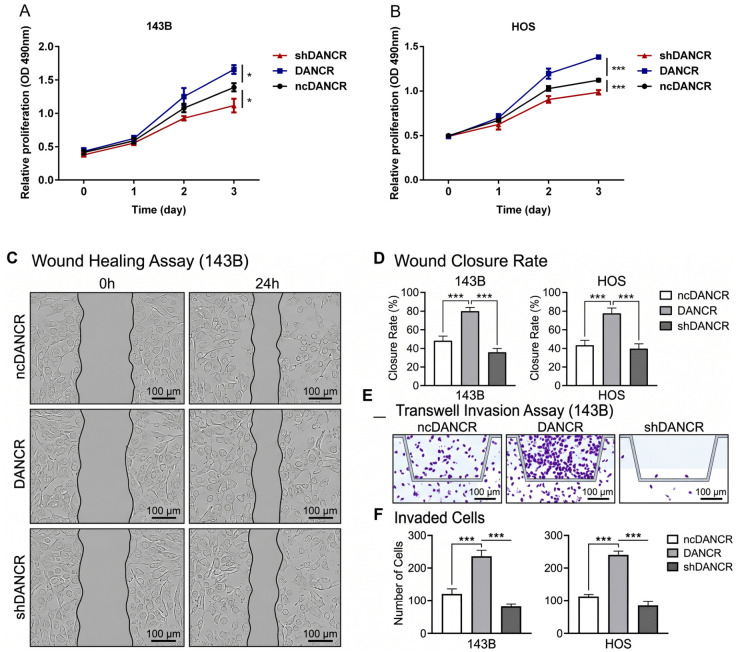
DANCR promotes the proliferation, migration, and invasion of 143B and HOS cells. (**A**,**B**) MTS proliferation assays for 143B (**A**) and HOS (**B**) cells at 0, 1, 2, and 3 days post-transfection. Cell proliferation was compared among cells transfected with the control vector (ncDANCR), DANCR overexpression vector (DANCR), and DANCR knockdown vector (shDANCR). (**C**) Representative cropped images of the wound-healing assay at 0 h and 24 h. The displayed panels are illustrative fields; wound closure was quantified from the entire matched wound area in the original paired 0 h and 24 h images, not from visual assessment of a single cropped field. The reference lines in the wound-healing panels indicate the wound edges at the corresponding time point. (**D**) Quantitative analysis of the wound-closure rate. (**E**) Representative images of invaded cells on the lower membrane surface after cotton-swab removal of non-invading cells from the upper surface of the Matrigel-coated membrane; stained cells visible in these panels represent lower-surface invaded cells. (**F**) Quantification of invaded cells counted from five random lower-surface microscopic fields per replicate. Data are shown as mean +/− SD from three independent experiments. * *p* < 0.05, *** *p* < 0.001.

The MTS assays showed that at 72 h, the A490 values of the *DANCR*-overexpressing cells were significantly higher than those of the control group, while the sh*DANCR* group showed significantly lower values (*p* < 0.01) (Figure 4A,B). For the wound-healing assay, ImageJ-based quantification was performed using the entire matched wound area in the original paired 0 h and 24 h images, rather than by visual estimation from the cropped representative panels; this analysis demonstrated that *DANCR* overexpression increased the wound-closure rate, whereas *DANCR* knockdown reduced wound closure (Figure 4C,D). In the Transwell invasion assay, only cells that had migrated through the Matrigel-coated membrane and remained attached to the lower membrane surface after removal of non-invading upper-surface cells were counted. Quantitative analysis of five random lower-surface fields per replicate indicated increased invasion after *DANCR* overexpression and decreased invasion after *DANCR* knockdown (Figure 4E,F). These results support a pro-proliferative and pro-motility effect of lncRNA-*DANCR* in osteosarcoma cells.

### 3.3. DANCR Promotes Osteosarcoma Growth and Metastasis In Vivo

**Figure 5:**
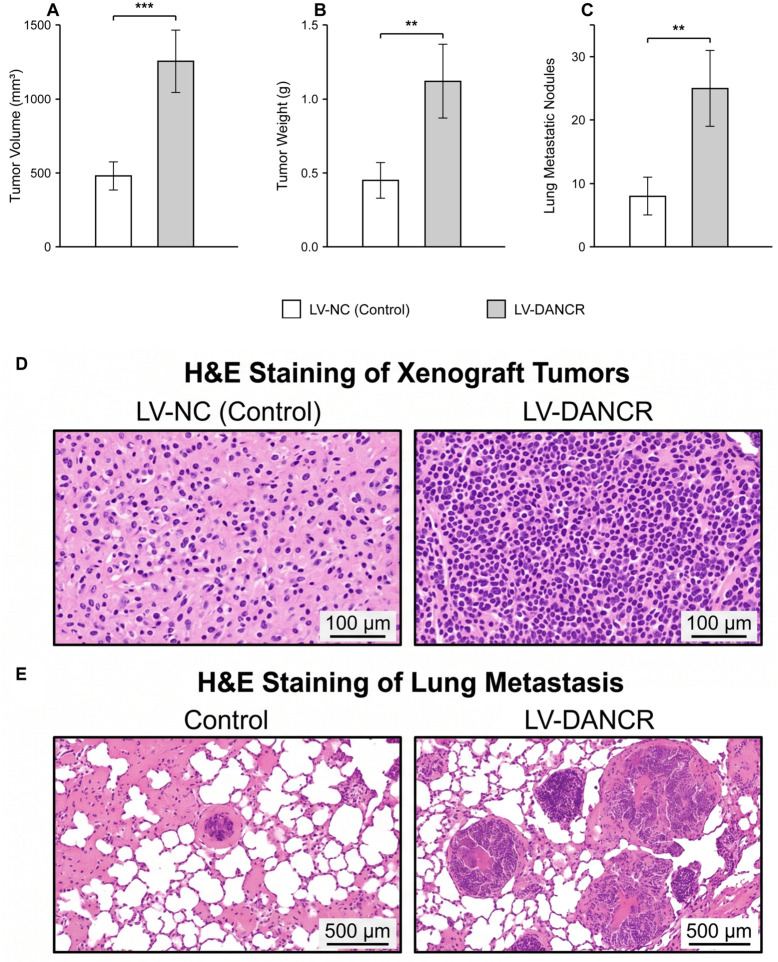
*DANCR* promotes osteosarcoma growth and metastasis in vivo. The effects of *DANCR* overexpression on tumor progression were assessed in a xenograft mouse model using 143B cells stably expressing LV-*DANCR* or a negative control vector (LV-NC). (**A**) Quantitative analysis of tumor volume measured on day 28 post-injection. (**B**) Quantitative analysis of final tumor weight after excision. (**C**) Quantification of lung metastatic nodules observed after 6 weeks. (**D**) Representative H&E staining images of the primary xenograft tumor tissues showing dense cellularity in the LV-*DANCR* group. (**E**) Representative H&E staining images of lung sections highlighting metastatic tumor deposits. Data are presented as the mean ± SD (*n* = 6 per group). Statistical significance was determined using Student’s *t*-test. ** *p* < 0.01, *** *p* < 0.001 vs. the control group.

To investigate the role of *DANCR* in vivo, we established a xenograft model using 143B cells stably overexpressing *DANCR*. As shown in Figure 5A, overexpression of *DANCR* led to a significant increase in tumor volume compared to the control group (1255 ± 210 mm^3^ vs. 480 ± 95 mm^3^; *p* < 0.001). Consistent with the tumor volume data, the final tumor weight was also substantially higher in the LV-*DANCR* group (Figure 5B, 1.12 ± 0.25 g vs. 0.45 ± 0.12 g; *p* < 0.01). Representative H&E staining showed densely cellular tumor areas in LV-*DANCR* xenografts, consistent with the increased tumor burden observed in vivo (Figure 5D). Furthermore, we assessed the effect of *DANCR* on metastatic potential by quantifying lung nodules. The number of metastatic nodules was significantly greater in mice from the LV-*DANCR* group than in the control group (Figure 5C, 25 ± 6 vs. 8 ± 3; *p* < 0.01). Representative H&E images of the resected lung tissues further demonstrated metastatic tumor deposits in the LV-*DANCR* cohort, supporting the macroscopic lung nodule counts (Figure 5E). Concurrent macroscopic and microscopic examinations of other major organs, including the liver, brain, and kidneys via standard H&E staining, revealed no obvious extra-pulmonary metastatic lesions within this 6-week timeframe.

To rigorously assess the actual tumor-initiation capacity rather than just passive proliferation, an in vivo limiting dilution assay was concurrently deployed. Subcutaneous injection of varied minute cell numbers conclusively highlighted that the *DANCR*-overexpressing cells harbored a significantly higher frequency of intrinsic tumor-initiating cells compared to their control counterparts (Table 2). Taken together, these comprehensive in vivo models definitively assert that *DANCR* fuels both the localized expansion and distant dissemination of osteosarcoma.

### 3.4. DANCR Up-Regulated CD133 by Competing for Binding to miR-145-5p and Activating the Akt Signaling Pathway

**Figure 6:**
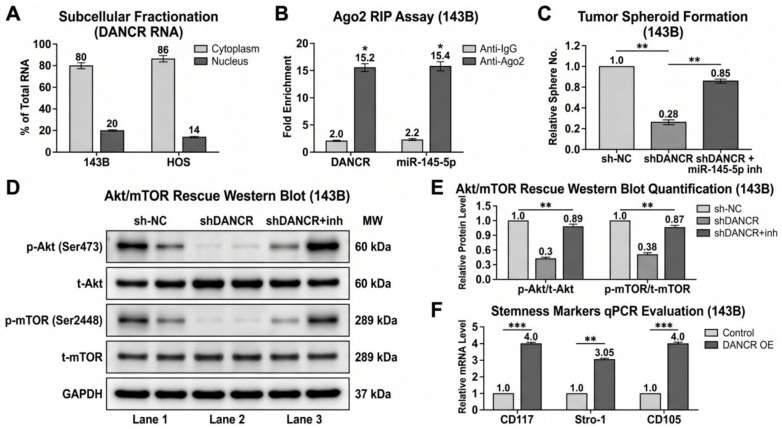
Mechanistic validation and rescue of the *DANCR*/*miR-145-5p* ceRNA network. (**A**) RT-qPCR analysis of *DANCR* expression in separated nuclear and cytoplasmic fractions of 143B and HOS cells, demonstrating predominant cytoplasmic localization. (**B**) Ago2 RNA immunoprecipitation (RIP) assay in 143B cells demonstrating the robust and significant enrichment of both *DANCR* and *miR-145-5p* in Ago2-immunoprecipitates compared to IgG controls. (**C**) Functional rescue of the tumor spheroid formation capacity in stable sh*DANCR* 143B cells following co-transfection with a specific *miR-145-5p* inhibitor. (**D**,**E**) Representative Western blot bands (**D**) and corresponding quantification (**E**) showing the restorative effect of the *miR-145-5p* inhibitor on the phosphorylation status (p-Akt/t-Akt and p-mTOR/t-mTOR ratios) that was previously suppressed by *DANCR* silencing. (**F**) RT-qPCR evaluation demonstrating significant upregulation of alternative established stemness markers CD117, Stro-1, and CD105 following stable *DANCR* overexpression. Data represent mean ± SD from three independent experiments. * *p* < 0.05, ** *p* < 0.01, *** *p* < 0.001.

To validate the ceRNA mechanism physically, we first confirmed the subcellular localization of *DANCR*. Subcellular fractionation followed by qRT-PCR revealed that *DANCR* was predominantly localized in the cytoplasm of both 143B and HOS cells, providing the spatial prerequisite for miRNA sponging (Figure 6A). Next, an Ago2 RNA immunoprecipitation (RIP) assay demonstrated a striking and specific enrichment of both *DANCR* and *miR-145-5p* transcripts in the Ago2 precipitates compared to the nonspecific IgG control (Figure 6B), confirming their direct physical association within the RNA-induced silencing complex (RISC).

To rigorously substantiate the proposed regulatory axis functionally, we performed rescue experiments. Strikingly, co-transfection of the stable sh*DANCR* cells with a specific *miR-145-5p* inhibitor successfully rescued the spheroid formation capacity that had been severely diminished by *DANCR* depletion (Figure 6C). Moreover, this miRNA inhibition successfully and effectively reversed the suppression of Akt and mTOR-specific phosphorylation induced by *DANCR* silencing, as evidenced by the restored protein levels and quantification of key pathway indicators (Figure 6D,E). We additionally explored the impact of *DANCR* on an expanded panel of established stemness markers and discovered that *DANCR* overexpression independently and significantly upregulated the mRNA transcript levels of CD117, Stro-1, and CD105 (Figure 6F).

**Figure 7:**
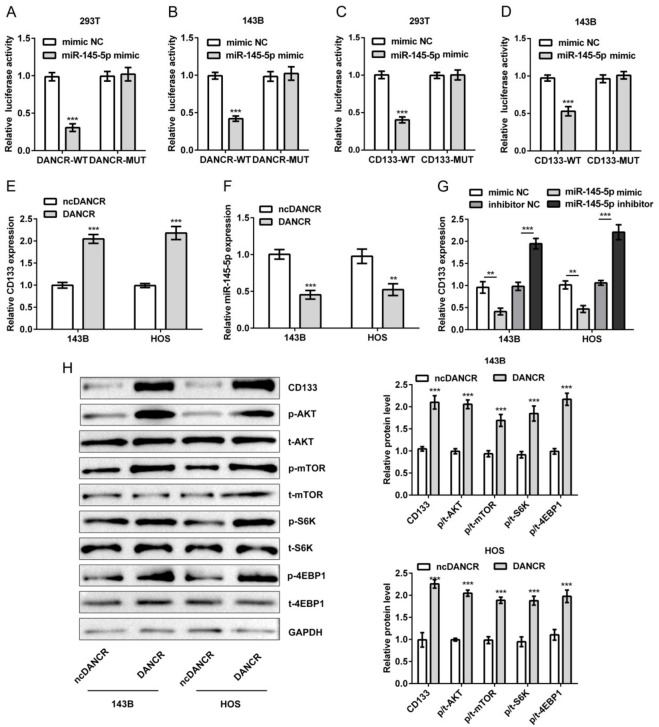
*DANCR* upregulates CD133 by competitively binding to *miR-145-5p* and activating the AKT signaling pathway. (**A**,**B**) Dual-luciferase reporter assays in 293T (**A**) and 143B (**B**) cells co-transfected with *DANCR* wild-type (WT) or mutant (MUT) reporters and *miR-145-5p* mimic or mimic NC. (**C**,**D**) Dual-luciferase reporter assays in 293T (**C**) and 143B (**D**) cells co-transfected with CD133-3′UTR-WT or -MUT reporters and *miR-145-5p* mimic or mimic NC. (**E**) qRT-PCR of CD133 mRNA expression and (**F**) *miR-145-5p* expression in 143B and HOS cells after *DANCR* overexpression. (**G**) qRT-PCR of CD133 mRNA in 143B and HOS cells treated with *miR-145-5p* mimic or inhibitor. (**H**) Western blot analysis and quantification of CD133, total (t-) and phosphorylated (p-) AKT, mTOR, S6K, and 4EBP1 in 143B and HOS cells overexpressing *DANCR*. GAPDH served as a loading control. Specific phosphorylation sites interrogated included AKT (Ser473), mTOR (Ser2448), S6K (Thr389), and 4EBP1 (Thr37/46). Data are shown as mean ± SD. ** *p* < 0.01, *** *p* < 0.001.

To further investigate the molecular binding, we constructed a luciferase reporter plasmid containing the wild-type *DANCR* sequence (*DANCR*-WT) harboring the predicted *miR-145-5p* binding site. These luciferase plasmids and *miR-145-5p* mimics were co-transfected into HEK293T cells and osteosarcoma cells 143B. The *miR-145-5p* mimetic reduced the luciferase activity of the *DANCR*-WT reporter vector by approximately 60%, but did not affect the activity of the reporter with a mutated binding site (*DANCR*-MUT) in HEK293T cells (Figure 7A). These results were confirmed in 143B cells (Figure 7B). To determine whether *miR-145-5p* also binds to the CD133 3′-UTR, a luciferase reporter gene containing the predicted binding site was constructed. Similarly, the *miR-145-5p* mimetic reduced the luciferase activity of the CD133-WT reporter vector, but did not reduce the luciferase activity of the CD133-MUT reporter vector in HEK293T cells (Figure 7C). This was confirmed in 143B cells (Figure 7D).

qRT-PCR explored the relationship between the expression levels of these genes, and CD133 mRNA was highly expressed in 143B and HOS cells overexpressing *DANCR*, compared with control cells (Figure 7E). Compared with control cells, *miR-145-5p* was significantly decreased in ectopic *DANCR*-transfected cells (Figure 7F). In addition, *miR-145-5p* inhibitor significantly increased CD133 mRNA expression, whereas the *miR-145-5p* mimic reduced CD133 mRNA expression in 143B and HOS cells (Figure 7G). The above results demonstrated that *DANCR* can competitively bind to *miR-145-5p* to block its function. Given previous reports that CD133 may function not merely as a surface marker but also as a signaling-associated receptor capable of interacting with the PI3K regulatory subunit, we performed molecular profiling by Western blot analysis. The results showed that elevated *DANCR* increased CD133 protein abundance and was accompanied by phosphorylation-dependent activation of downstream PI3K/Akt-related markers, including increased p-AKT (Ser473), p-mTOR (Ser2448), p-S6K (Thr389), and p-4EBP1 (Thr37/46) levels compared with the control cells (Figure 7H). Although these data support a link between *DANCR*-induced CD133 upregulation and Akt/mTOR pathway activation, further CD133 loss-of-function rescue experiments will be required to prove that CD133 is indispensable for this signaling effect.

### 3.5. DANCR Modulates miR-145-5p-Related Changes in Autophagy-Associated Markers in 143B Cells

**Figure 8:**
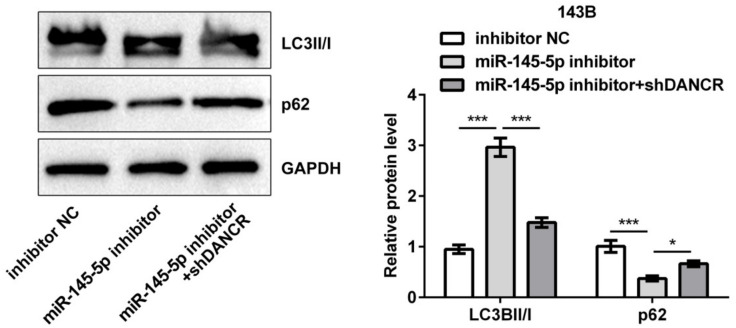
The *DANCR*/*miR-145-5p* axis modulates autophagy-associated markers in 143B cells. Representative Western blot (**left**) and quantification (**right**) of the LC3B-II/I ratio and p62 protein levels in 143B cells transfected with inhibitor NC, *miR-145-5p* inhibitor, or *miR-145-5p* inhibitor combined with sh*DANCR*. GAPDH was used as a loading control. Data are shown as mean ± SD. * *p* < 0.05, *** *p* < 0.001.

Because autophagy regulation in cancer cells is context-dependent and may not be fully inferred from single Akt/mTOR readouts, we examined whether the *DANCR*/*miR-145-5p* axis altered autophagy-associated markers in 143B cells. We assessed the levels of microtubule-associated protein 1A/1B-light chain 3-II (LC3B-II), a marker associated with autophagosome formation, and sequestosome-1 (p62/SQSTM1), a protein degraded during autophagy [16]. Compared with the control group, the LC3B-II/I ratio was significantly increased in the *miR-145-5p* inhibitor group (2.96 ± 0.15, *p* < 0.001), and the expression of p62 was significantly decreased (0.37 ± 0.04, *p* < 0.001), indicating changes in autophagy-associated markers. In the *miR-145-5p* inhibitor plus sh*DANCR* group, the LC3B-II/I ratio was decreased (1.48 ± 0.08, *p* < 0.001) and p62 was increased (0.66 ± 0.05, *p* < 0.05), compared with the *miR-145-5p* inhibitor group (Figure 8). These results indicate that *DANCR* depletion counteracts the LC3B-II/I and p62 changes induced by *miR-145-5p* inhibition. However, because LC3B-II and p62 immunoblotting alone cannot distinguish altered autophagosome formation from altered degradation, these findings should be interpreted as changes in autophagy-associated markers rather than definitive evidence of altered autophagic flux.

## 4. Discussion

Despite their lack of translational capacity, lncRNAs represent a massive and dynamically regulated segment of the genomic transcriptome. Mounting evidence unequivocally places them at the epicenter of oncogenesis, where they delicately govern the aggressive progression and metastatic escape of sarcomas, as classically exemplified by transcripts such as MALAT1 and TUG1 [18,19]. Historically, the intricate mechanism by which lncRNAs modulate upstream receptor networks, particularly in solid bone tumors, has remained obscure. The current study showed that *DANCR* was upregulated in osteosarcoma tissues and positively correlated with stemness-related markers, suggesting a potential association between *DANCR* dysregulation and aggressive tumor biology. Phenotypically, exogenous *DANCR* overexpression forcefully accelerated osteosarcoma cell proliferation, migration, and in vitro invasion, while targeted *DANCR* depletion efficiently paralyzed these malignant traits. Strikingly, ectopic *DANCR* expression concurrently exacerbated robust xenograft tumor outgrowth and rampant pulmonary dissemination in vivo.

Mirroring the functional properties of established master regulators [18,19,20], our clinical screening disclosed a profound correlation between endogenous *DANCR* levels and core stemness indices. Utilizing established cellular models, we determined that ectopic *DANCR* not only boosted the transcriptional output of CD133, SOX2, and CD90 but crucially expanded the elusive CD133+/CD44+ cellular fraction. Although marker-defined CD133+/CD44+ populations may vary according to cell-line background and gating strategy, the consistent increase in this fraction, together with enhanced spheroid formation and limiting dilution results, supports a *DANCR*-associated expansion of stem-like phenotypes. Decades of lineage-tracing studies confirm that even minor expansions in true CSC pools dictate overwhelming therapeutic resistance and explosive systemic relapses [21,22]. Consequently, the *DANCR*-driven enlargement of the tumor sphere compartment intrinsically enhances generalized tumorigenesis.

Deciphering the spatial mechanics of lncRNAs is mandatory for understanding their functional spectrum. While nuclear-retained transcripts dictate chromosomal architecture, those transported to the cytoplasm invariably function as post-transcriptional modulators, predominantly serving as decoys for miRNAs [23,24]. Crucially, our newly supplemented fractionation data definitively authenticated that *DANCR* resides predominantly within the cellular cytoplasm, perfectly aligning with its presumed ceRNA vocation. As an indispensable surface architect of the stem cell niche, CD133 directly orchestrates malignant phenotypes and intractable chemoresistance across diverse cancers, including osteosarcoma [25,26,27]. Because the canonical PI3K/Akt/mTOR signaling axis relentlessly dictates survival and metabolic advantages [28], mapping the regulatory connection between *DANCR* and CD133 is clinically imperative. Fascinatingly, our rigorous validation assays, including RIP and reporter systems, authenticated a ceRNA network where *DANCR* and the CD133 3′-UTR competitively sponge the tumor-suppressive *miR-145-5p*. Furthermore, CD133 has recently been characterized as an active signaling receptor. Recent comprehensive updates robustly reinforce that the intracellular domain of CD133 directly interacts with the PI3K p85 regulatory subunit, thereby potently initiating the downstream Akt kinase cascade [29,30].

The paradigm we uncovered is consistent with a ceRNA mechanism in which *DANCR* sequesters *miR-145-5p* and thereby relieves repression of CD133. Furthermore, the *DANCR*/*miR-145-5p*/CD133 axis was associated with Akt/mTOR pathway activation and with changes in LC3B-II/I and p62 levels. However, because these autophagy-associated changes do not fully establish a simple Akt/mTOR-dependent flux model, additional flux-based assays using lysosomal inhibitors such as chloroquine or bafilomycin A1 will be required to define the precise causal relationship. Thus, our data support an association between the *DANCR* sponge network, stemness rescue, and autophagy-related marker changes, while the mechanistic boundaries of this metabolic dependency should be tested in future orthogonal studies.

## 5. Conclusions

In summary, our study suggests that *DANCR* promotes osteosarcoma stemness and progression at least partly through a *DANCR*/*miR-145-5p*/CD133 regulatory axis. Subcellular fractionation, Ago2-RIP, rescue experiments, and in vivo limiting dilution data strengthen the evidence that this axis contributes to stem-like phenotypes, tumor-initiating capacity, and Akt/mTOR pathway activation. Nevertheless, the causal requirement of CD133 for *DANCR*-induced Akt/mTOR activation and the precise regulation of autophagic flux require further validation. These findings support the *DANCR*/*miR-145-5p*/CD133 axis as a potential molecular vulnerability in osteosarcoma and provide a basis for future mechanistic and therapeutic studies. These findings are summarized in the proposed schematic model (Graphical Abstract).

## Figures and Tables

**Table 1:** Detailed information of primary antibodies utilized in Western blotting.

Target Protein	Company (Origin)	Catalog Number	Application/Dilution
CD133	Cell Signaling Technology (Danvers, MA, USA)	#64326	WB (1:1000)
Total AKT	Cell Signaling Technology (Danvers, MA, USA)	#4691	WB (1:1000)
Phospho-AKT (Ser473)	Cell Signaling Technology (Danvers, MA, USA)	#4060	WB (1:1000)
Total mTOR	Cell Signaling Technology (Danvers, MA, USA)	#2983	WB (1:1000)
Phospho-mTOR (Ser2448)	Cell Signaling Technology (Danvers, MA, USA)	#5536	WB (1:1000)
Total p70S6K	Cell Signaling Technology (Danvers, MA, USA)	#2708	WB (1:1000)
Phospho-p70S6K (Thr389)	Cell Signaling Technology (Danvers, MA, USA)	#9234	WB (1:1000)
Total 4EBP1	Cell Signaling Technology (Danvers, MA, USA)	#9644	WB (1:1000)
Phospho-4EBP1 (Thr37/46)	Cell Signaling Technology (Danvers, MA, USA)	#2855	WB (1:1000)
LC3B	Cell Signaling Technology (Danvers, MA, USA)	#3868	WB (1:1000)
p62/SQSTM1	Cell Signaling Technology (Danvers, MA, USA)	#5114	WB (1:1000)
GAPDH	Cell Signaling Technology (Danvers, MA, USA)	#5174	WB (1:5000)

**Table 2:** In vivo limiting dilution assay of tumor-initiating capacity in stably transfected 143B xenograft models.

Cell Line Group	Injected Cell Dose	Tumor Incidence (Positive/Total)	Estimated Stem Cell Frequency (95% CI)	*p*-Value
143B (LV-NC)	1 × 10^5^	5/6	1/85,340 (1/39,400 to 1/184,800)	-
	1 × 10^4^	1/6		
	1 × 10^3^	0/6		
143B (LV-*DANCR*)	1 × 10^5^	6/6	1/12,450 (1/5600 to 1/27,600)	<0.001
	1 × 10^4^	4/6		
	1 × 10^3^	1/6		

## Data Availability

The original contributions presented in this study are included in the article. Further inquiries can be directed to the corresponding author.

## Abbreviations

ceRNA	Competing endogenous RNA
CSC	Cancer stem cell
*DANCR*	Differentiation antagonizing non-protein-encoding RNA
DFS	Disease-free survival
EMT	Epithelial–mesenchymal transition
FACS	Fluorescence-activated cell sorting
LC3B	Microtubule-associated protein 1A/1B-light chain 3
lncRNA	Long noncoding RNA
miRNA	MicroRNA
MUT	Mutant
NC	Negative control
OS	Overall survival
p62/SQSTM1	Sequestosome-1
qRT-PCR	Quantitative real-time polymerase chain reaction
STR	Short tandem repeat
TIC	Tumor-initiating cell
WT	Wild-type
